# Remarks on the Treatment to Be Adopted during Labour in Cases of the Anterior Obliquity of the Uterus

**Published:** 1827-09

**Authors:** John North

**Affiliations:** Surgeon Accoucheur.


*Remarks on the Treatment to be adopted during Labour in Cases of the anterior Obliquity of the Uterus.* By JOHN NORTH, Esq. Surgeon Accoucheur.

THE natural position of the os uteri at the commencement of labour, is when it is placed near the centre of the superior aperture of the cavity of the pelvis: but it rarely happens that the os uteri is thus favourably situated. The fundus of the uterus generally inclines either to the left or right side, or it falls forwards. In whichever of these situations the fundus may be placed, the mouth of the uterus must be thrown from the axis of the pelvis, and the sufferings of the woman will be protracted during labour. It is not my intention to enter into a formal consideration of all the various displacements of the uterus which may occasionally occur: I shall confine my observations to the anterior obliquity of the uterus, which is sometimes a source of much inconvenience during pregnancy, as well as in the act of parturition. When the fundus of the uterus falls very far forward, the common centre of gravity of the patient is destroyed, and the woman walks with difficulty, from the exertion she is under the necessity of making to preserve her equilibrium. From such a displacement of the uterus, she will also suffer from pain in the back and loins, together with frequent inclination to make water or to go to stool. In some cases it may be necessary that the patient should remain in a recumbent position, to alleviate these distressing symptoms. A proper bandage, however, if adroitly applied, will generally afford the desired relief. The most convenient and effectual bandage for this purpose has been described by Dr. DEWEES.

When this anterior obliquity of the uterus exists, the os uteri must necessarily be thrown backward in a degree corresponding with the falling forwards of the fundus; and in many instances the practitioner will be unable, by an ordinary examination, to determine the situation of the os uteri for many hours after labour has commenced. In such a case there are two modes of practice we may adopt: we may either depend upon the uterine efforts to place the parts in a more favourable situation, and thus terminate the labour without our assistance; or we may introduce the hand into the vagina, and, with a finger hooked within the os uteri, endeavour to draw it towards the centre of the inferior aperture of the pelvis. Dr. Dewees observes,* that, "whenever it is found that the os uteri cannot be reached by a well-directed search in the ordinary way, we must introduce the hand, well lubricated, so that its palm may be next to the distended uterus. A finger should then be made to reach up to the neighbourhood of the projection of the sacrum, whereon some one portion of the uterine globe, the os uteri will be detected: when discovered, we should hook it upon the point of the finger, (provided it is either dilated or easily dilatable,) and draw it towards the centre of the inferior strait. When it has followed so far, the hand may be gently withdrawn, (but not the finger from the os uteri,) and the uterus detained there until the proper direction of the forces and the axis of the uterus are made to correspond."

That some labours are procrastinated by the mere oblique position of the os uteri, I do not doubt; but, as far as I can judge, the manual interference recommended by Dr. Dewees is not necessary, and consequently injudicious, in the great majority of cases. Dr. DENMAN was of opinion that when the oblique position of the os uteri does retard a labour, or accompany a difficult one, that it does not require any manual assistance, or that we should retract it to a central position with respect to the cavity of the pelvis. He conceived that the difficulty would be overcome by placing the patient in a proper situation. In cases of anterior obliquity of the uterus, she should be placed upon her back, and the fundus of the uterus supported by an assistant. If the opinion I have formed upon this subject had not been countenanced by the high authority of Dr. Denman, I might have hesitated to oppose the doctrine laid down by so experienced and able a practitioner as Dr. Dewees. I have known many instances where the os uteri was thrown so far backward, in consequence of the displacement of the uterus to which I have referred, that its situation could not be determined for some time after the commencement of labour. But I have seen no case in which the unassisted efforts of nature have not terminated the labour in a favourable manner; and I should con-

* System of Midwifery, p. 89, Lond. ed.

sequently be inclined to deprecate the conduct of the practitioner who would, in every case, if he could not reach the os uteri in the ordinary way, introduce his hand, and endeavour to "hook" down the os uteri. According to the direction of Dr. Dewees, we are not to remove the finger from the os uteri "until the proper direction of the forces and the axis of the uterus are made to correspond." It must obviously, then, be impossible for the practitioner to determine whether the favourable change in the position of the parts is the result of his assistance, or of the continued uterine efforts which have been going on.

In some instances I have known inflammation of the uterus produced by the rough and repeated efforts of young practitioners to "hook" down the os uteri; and, as I believe that nothing more is generally required in such cases than moderate patience, I may be allowed to enter my humble protest against the practice recommended by Dr. Dewees, although I have no doubt *he* would have recourse to it without injury to the patient.

But, however desirable it may be that the process of labour should not be improperly interfered with, it is equally necessary that we should not withhold our aid when it is really required. If it should happen that the uterine efforts do not gradually bring the os uteri into the direction of the axis of the inferior aperture, and that the head of the child is forced down by each pain, with the body of the uterus expanded over it, it would then be proper for the practitioner to endeavour to place the parts in a more favourable situation, by the practice recommended by Dr. Dewees: I apprehend, however, that such cases are very rare.

*Upper Berkeley-street, Portman-square;*
*August* 1827.

